# Pharmaceutical care in Brazil’s primary health care

**DOI:** 10.11606/S1518-8787.2017051007109

**Published:** 2017-09-22

**Authors:** Patricia Sodré Araújo, Ediná Alves Costa, Augusto Afonso Guerra, Francisco de Assis Acurcio, Ione Aquemi Guibu, Juliana Álvares, Karen Sarmento Costa, Margô Gomes de Oliveira Karnikowski, Orlando Mario Soeiro, Silvana Nair Leite

**Affiliations:** I Departamento de Ciências da Vida. Curso de Farmácia. Universidade do Estado da Bahia. Salvador, BA, Brasil; II Instituto de Saúde Coletiva. Universidade Federal da Bahia. Salvador, BA, Brasil; III Departamento de Farmácia Social. Faculdade de Farmácia. Universidade Federal de Minas Gerais. Belo Horizonte, MG, Brasil; IV Faculdade de Ciências Médicas. Santa Casa de São Paulo. São Paulo, SP, Brasil; V Núcleo de Estudos de Políticas Públicas. Universidade Estadual de Campinas. Campinas, SP, Brasil; VI Programa de Pós-Graduação em Saúde Coletiva. Departamento de Saúde Coletiva. Faculdade de Ciências Médicas. Universidade Estadual de Campinas. Campinas, SP, Brasil; VII Programa de Pós-Graduação em Epidemiologia. Faculdade de Medicina. Universidade Federal do Rio Grande do Sul. Porto Alegre, RS, Brasil; VIII Faculdade de Ceilândia. Universidade de Brasília. Brasília, DF, Brasil; IX Faculdade de Ciências Farmacêuticas. Pontifícia Universidade Católica de Campinas. Campinas, SP, Brasil; X Departamento de Ciências Farmacêuticas. Universidade Federal de Santa Catarina. Florianópolis, SC, Brasil

**Keywords:** Pharmacists, Pharmaceutical Care, Pharmaceutical Services, Primary Health Care, Health Services Research, Unified Health System, Farmacêuticos, Atenção Farmacêutica, Assistência Farmacêutica, Atenção Primária à Saúde, Pesquisa sobre Serviços de Saúde, Sistema Único de Saúde

## Abstract

**OBJECTIVE:**

To characterize the activities of clinical nature developed by pharmacists in basic health units and their participation in educational activities aiming at health promotion.

**METHODS:**

This article is part of the *Pesquisa Nacional sobre Acesso, Utilização e Promoção do Uso Racional de Medicamentos* – *Serviços, 2015* (PNAUM – National Survey on Access, Use and Promotion of Rational Use of Medicines – Services, 2015), a cross-sectional and exploratory study, of evaluative nature, consisting of a survey of information in a representative sample of cities, stratified by the Brazilian regions that constitute domains of study, and a subsample of primary health care services. The interviewed pharmacists (n=285) were responsible for the delivery of medicines and were interviewed in person with the use of a script. The characterization of the activities of clinical nature was based on information from pharmacists who declared to perform them, and on participation in educational activities aiming at health promotion, according to information from all pharmacists. The results are presented in frequency and their 95% confidence intervals.

**RESULTS:**

From the interviewed subjects, 21.3% said they perform activities of clinical nature. Of these, more than 80% considered them very important; the majority does not dispose of specific places to perform them, which hinders privacy and confidentiality in these activities. The main denominations were “pharmaceutical guidance” and “pharmaceutical care.” The registration of activities is mainly made in the users’ medical records, computerized system, and in a specific document filed at the pharmacy, impairing the circulation of information among professionals. Most pharmacists performed these activities mainly along with physicians and nurses; 24.7% rarely participated in meetings with the health team, and 19.7% have never participated.

**CONCLUSIONS:**

Activities of clinical nature performed by pharmacists in Brazil are still incipient. The difficulties found point out to the professionals’ improvisation and effort. The small participation in educational activities of health promotion indicates little integration of pharmacists with the health team and of pharmaceutical services with other health actions.

## RESUMO

**OBJETIVO:**

Caracterizar as atividades de natureza clínica desenvolvidas pelos farmacêuticos nas unidades básicas de saúde e sua participação em atividades educativas de promoção da saúde.

**MÉTODOS:**

O artigo integra a Pesquisa Nacional sobre Acesso, Utilização e Uso Racional de Medicamentos – Serviços 2015, estudo transversal, exploratório, de natureza avaliativa, composto por levantamento de informações numa amostra representativa de municípios, estratificada pelas regiões do Brasil que constituem domínios do estudo, e sub-amostra de serviços de atenção básica. Os farmacêuticos entrevistados (n = 285) eram responsáveis pela entrega de medicamentos e foram entrevistados pessoalmente com uso de roteiro. A caracterização das atividades de natureza clínica baseou-se em informações dos farmacêuticos que declararam realizá-las, e a participação em atividades educativas e voltadas à promoção da saúde, em informações de todos os farmacêuticos. Os resultados são apresentados em frequência e respectivos intervalos de confiança de 95%.

**RESULTADOS:**

Dos entrevistados, 21,3% afirmaram realizar atividades de natureza clínica. Destes, mais de 80% as consideram muito importante; a maioria não dispõe de local específico para realizá-las, prejudicando a privacidade e confidencialidade nessas atividades. As principais denominações foram orientação farmacêutica e atenção farmacêutica. O registro das atividades é feito principalmente em prontuário do usuário, sistema informatizado e documento próprio arquivado na farmácia, o que dificulta a circulação das informações entre os profissionais. A maioria realiza as atividades principalmente em conjunto com médicos e enfermeiros; 24,7% raramente participam de reuniões com a equipe de saúde e 19,7% nunca participou.

**CONCLUSÕES:**

As atividades de natureza clínica desempenhadas por farmacêuticos no Brasil ainda são incipientes. As dificuldades encontradas apontam improvisação e esforço dos profissionais. A pequena participação em atividades educativas de promoção da saúde indica pouca integração dos farmacêuticos na equipe de saúde e da assistência farmacêutica nas demais ações de saúde.

## INTRODUCTION

Drug-related morbidity and mortality has been pointed out as an important public health problem around the world^6^ ^,^ ^14^ ^,^ ^17^. In Brazil, studies have shown the impact on society and on the Brazilian Unified Health System (SUS)^3^ ^,^ ^25^. Among the drug-related problems, it is possible to highlight those related to indication, effectiveness, safety, and adherence. These problems cause morbidity and mortality related to these technologies, which result in high social impact within the clinical, humanistic, and economic contexts. The main risk factors associated with the occurrence of these problems include age, presence of comorbidities, and polipharmacotherapy^10^ ^,^ ^14^ ^,^ ^25^. Preventing and minimizing this impact require actions that interfere in the process of use of medicines by users, by managing the drug therapy^6^ ^,^ ^17^. These are important challenges of the current pharmaceutical services agenda in Brazil.

In several countries^5^ ^,^ ^7^ ^,^ ^9^ ^,^ ^21^ ^,^ ^23^ ^,^ ^27^, pharmaceutical care^a^ has been assumed as strategic policy for reducing the impact of drug-related morbidity and mortality ^6^ ^,^ ^12^ ^,^ ^17^. In Brazil, it is part of the *Política Nacional de Assistência Farmacêutica* (PNAF – National Policy of Pharmaceutical Services). However, research on this subject are scarce, even in studies that discuss pharmaceutical policies that prescribe these practices in their devices, as an example of the specialized component of pharmaceutical services. The studies that investigate such activities take place in SUS and their results are also incipient.

^a^ Pharmaceutical care in the National Policy of Pharmaceutical Services is defined as an action of pharmaceutical services, considered a model of the practice performed by the pharmacist. In this policy, the *modus operandi* of this practice includes the direct interaction of the pharmacist with the user, with the objective of providing a rational pharmacotherapy and obtaining of defined and measurable clinical results, in addition to understanding this practice as important to the completeness of the health actions.

The subject of pharmaceutical care finds some difficulties, especially regarding the understanding of its meaning. Worldwide, the definition adopted by Hepler and Strand^10^ (1990) is considered a consensual milestone. However, in Brazil, what is called pharmaceutical care assumes different meanings in what could be called “world of science” and in how the policies in the field of pharmaceutical services understand it. The differences are in the epistemological plan and are repeated during practice.

The names that have been widely used among pharmacists to designate services of clinical nature are: cognitive pharmaceutical services, pharmaceutical consultation, pharmaceutical guidance, patient education, clinical pharmacy, and pharmaceutical care/drug therapy follow-up/management of drug therapy. In several countries, the designation adopted for activities of clinical nature, the way they are performed, and the organizational aspects translate (or not) the elements that reveal the philosophical basis, the *modus operandi*, and the management aspects of these activities^17^ ^,^ ^19^.

Beyond the “cloudy weather”^17^ that involves what is called pharmaceutical care, in this study we understand as pharmaceutical activity of clinical nature all activities executed directly with the user and that has the purpose to meet the patients’ needs regarding the use of medicines and to express philosophical elements. According to Cipolle, Strand, and Morley^6^ (p. 73), “*the philosophy of pharmaceutical care includes several elements*. *It starts with the affirmation of a social necessity; it continues with a patient-centered approach to meet this need; it has as central element the care to another person by the development and maintenance of a therapeutic relationship, and concludes with a description of the professional’s actual responsibilities*.” Furthermore, a process of formal care, i.e., the *modus operandi* of care and management aspects.

There are, in Brazil, specific pharmaceutical policies that attribute to the pharmacist the exercise of activities of clinical nature – named in PNAF as pharmaceutical care. In addition, one can observe gaps regarding information on the performance or not of these activities in the primary care in SUS. Considering this, this study aimed to characterize the activities of clinical nature developed by pharmacists in basic health units of SUS and their participation in educational activities aiming at health promotion.

## METHODS

This study is part of the *Pesquisa Nacional sobre Acesso, Utilização e Promoção do Uso Racional de Medicamentos* – *Serviços, 2015* (PNAUM – National Survey on Access, Use and Promotion of Rational Use of Medicines – Services, 2015), whose goal was to characterize the organization of pharmaceutical services in the primary health care of SUS, aiming to promote the access and rational use of medicines, as well as to identify and discuss factors that interfere in the consolidation of pharmaceutical services in the municipal scope.

PNAUM is a cross-sectional exploratory study, with evaluative nature, consisting of a survey of information in a representative sample of primary health care services in representative cities in the regions of Brazil. Several populations were considered in the sampling, with samples stratified by the regions that constitute the domains of study. Face-to-face interviews were carried out with users, physicians, and those responsible for the delivery of medicines in the primary health care services of SUS, in addition to observation of pharmaceutical services and telephone interviews with the professionals responsible for pharmaceutical services and with the public administrators of the selected cities. The methodology of PNAUM, as well as the sampling process, are described in detail by Álvares et al.^1^

In this article, only pharmacists were selected among those responsible for the delivery of medicines. This is a descriptive and exploratory study with selected variables.

The characterization of pharmaceutical activities of clinical nature occurred based on the information provided by pharmacists who informed to perform this activity when answering the following question: “Do you perform any activity of clinical character?” From the affirmative to this question, we sought to identify the names and the importance attributed by pharmacists who declared to carry out the activity; among the others, the reasons for not carrying out those activities.

The relationship of the names of the activities of clinical nature included in the interview scripts considered those most used in publications of literature review: pharmaceutical care, drug therapy follow-up, clinical pharmacy, pharmaceutical consultation, pharmaceutical guidance, pharmaceutical assistance. The script also had a space for “other names,” which were little used and were not analyzed in this article. Furthermore, we included questions aiming to set up the presence of philosophical elements^6^ ^,^ ^16^ of the mentioned activity and of the process of care provided to the user. In addition, elements of a management system with human and material resources were organized, necessary to carry out the activities.

Considering the different conceptions of activities of clinical nature^2^ ^,^ ^6^ ^,^ ^17^, the participation in educational activities/health promotion activities was sought, as they are activities of diverse nature of dispensation or management functions specific to pharmaceutical activities.

The data were analyzed using the software SPSS® version 21. The analyses considered the sampling weights and the structure of the analysis plan for complex samples. A descriptive analysis of the variables used in the study was performed, presented according to the regions of Brazil with a 95% confidence interval.

PNAUM was approved by the National Research Ethics Committee, by Opinion no. 398.131/2013. All participants signed an informed consent form.

## RESULTS

We interviewed 1,139 individuals responsible for the delivery of medicines, of which 285 were pharmacists (32.7%); 106, pharmacy assistants (10.6%); 141, nurses (11.5%); 115, nursing technicians (8.3%); and 492 reported other professions or occupations (37%). From all the interviewed pharmacists, 79 (21.4%) said they perform activities of clinical nature.

Among the 285 pharmacists interviewed, more than 3/4 were aged between 30 and 59 years; most were women (except in the Midwest); and most had *latu sensu* graduate level (specialization addressed to professional practice), with the highest percentage in the Northeast (Table 1).

**Table 1:** Sociodemographic characteristics of pharmacists of primary health care according to the regions of Brazil. National Survey on Access, Use and Promotion of Rational Use of Medicines, 2015. (n = 285)

Variable	North	Northeast	Midwest	Southeast	South	Brazil
					
	% (IC95%)	% (IC95%)	% (IC95%)	% (IC95%)	% (IC95%)	% (IC95%)
Sex						
Female	68.5 (54.9–79.6)	69.6 (29.1–92.7)	49.9 (29.1–70.7)	60.9 (40.9–77.8)	84.8 (70.1–92.9)	64.6 (50.8–76.3)
Male	31.5 (20.4–45.1)	30.4 (7.3–70.9)	50.1 (29.3–70.9)	39.1 (22.2–59.1)	15.2 (7.1–29.9)	35.4 (23.7–49.2)
Skin color						
White	65.2 (50.1–77.8)	79.6 (45.2–94.8)	77.6 (61.8–88.1)	82.6 (69.9–90.6)	93.5 (77.4–98.3)	82.8 (74.7–88.6)
Black	–	11.3 (1.6–49.8)	1.4 (0.3–6.2)	0.7 (0.2–2.0)	–	1.5 (0.6–4.2)
Yellow	–	–	–	1.7 (0.3–9.5)	–	1.0 (0.2–6.4)
Mixed race	33.6 (21.1–48.8)	9.1 (4.2–18.7)	21.0 (10.7–37)	15.1 (7.3–28.4)	6.5 (1.7–22.6)	14.6 (9.1–22.6)
Indigenous	1.2 (0.2–8.3)	–	–	–	–	0.1 (0–0.4)
Marital status						
Single	32.7 (17.8–52.0)	10.0 (1.2–50.8)	61.1 (39.6–79.0)	32.2 (20.1–47.2)	37.7 (20.2–59.2)	34.1 (24.9–44.6)
Married	59.2 (40.8–75.3)	47.6 (9.5–88.7)	37.5 (19.7–59.5)	57.9 (41.7–72.6)	55.7 (34.3–75.1)	54.7 (42.7–66.1)
Stable union	7.0 (2.4–18.2)	1.9 (0.2–13.3)	0.2 (0–1.7)	4.7 (1.0–19.0)	4.4 (1.2–15.2)	4.0 (1.3–11.6)
Divorced	1.2 (0.2–8.3)	40.6 (5.5–89)	1.2 (0.2–8.5)	4.2 (2.0–8.6)	2.2 (0.3–12.7)	6.6 (2.3–17.9)
Widower	–	–	–	1.0 (0.2–4.4)	–	0.6 (0.1–2.9)
Age group (years)						
18 - 29	36.0 (22–52.9)	9.0 (0.9–51.7)	36.3 (17.6–60.2)	23.8 (12.8–39.9)	19.7 (8.9–38)	23.6 (15.6–34.1)
30 - 59	64.0 (47.1–78)	91.0 (48.3–99.1)	58.9 (34.3–79.6)	74.1 (58.3–85.4)	76.5 (56.8–88.9)	74.0 (63.5–82.3)
60 or more	–	–	4.9 (0.6–29.5)	2.1 (0.8–5.8)	3.9 (0.5–24.3)	2.4 (1.0–5.5)
Education level						
*Lato sensu* Specialization	38.3 (24.9–54.6)	65.7 (17.3–94.6)	35.1 (14.9–62.5)	35.2 (21.5–51.8)	51.2 (29.9–72.1)	40.4 (29.4–52.6)
Master’s or Doctorate degree	2.4 (0.6–9.2)	–	7.3 (1.6–27.2)	12.6 (2–50.2)	2.5 (0.4–12.3)	9.0 (1.8–34.2)

Source: PNAUM – Services, 2015.

Table 2 shows that, of the total pharmacists interviewed, just over 1/5 stated performing some activity of clinical nature. The highest percentage was achieved in the Northeast region (47.5%) and the lowest in the South (6.0%).

**Table 2:** Performance of activities of clinical nature, name, and importance given by pharmacists of primary health care, according to the regions of Brazil. National Survey on Access, Use and Promotion of Rational Use of Medicines, 2015.

Dimension/Variable	North	Northeast	Midwest	Southeast	South	Brazil
					
	% (IC95%)	% (IC95%)	% (IC95%)	% (IC95%)	% (IC95%)	% (IC95%)
Activities of clinical nature						
Perform them (n = 79)	29.8 (18.3–44.8)	47.5 (11.1–86.8)	20.2 (5.9–50.8)	21.2 (12.3–33.9)	6.0 (1.5–21.5)	21.3 (13.6–31.9)
Do not perform them (n = 206)	69.7 (55.2–81.7)	52.5 (13.2–88.9)	79.8 (49.2–94.1)	78.8 (66.1–87.7)	94.0 (78.5–98.5)	78.6 (68.1–86.4)
Reasons for not performing them (n = 206)						
Do not possess the necessary physical space	56.0 (37.0–73.4)	82.8 (29.3–98.2)	56.3 (28.4–80.7)	42.1 (24.4–62.1)	43.7 (23.3–66.5)	46.7 (33.4–60.5)
Do not have enough time	49.1 (29.9–68.7)	19.0 (2.3–70.5)	58.6 (36.8–77.5)	50.6 (30.9–70)	28.7 (15.9–46.2)	45.4 (32.0–59.6)
Was never asked to perform them	44.1 (26.0–64.0)	84.4 (66.9–93.6)	58.1 (30.5–81.4)	49.9 (29.9–69.9)	52.7 (30.1–74.3)	53.0 (38.6–67.0)
Other	7.9 (1.8–28.4)	–	15.2 (5.6–35.2)	7.4 (2.7–18.9)	21.3 (9.2–41.8)	10.4 (5.8–17.8)
Naming (n = 79)						
Pharmaceutical care	61.4 (36.2–81.7)	2.0 (0.1–21.6)	29.4 (5.3–75.8)	45.8 (23.2–70.3)	35.4 (4.3–87.1)	36.2 (18.5–58.7)
Drug therapy follow-up	3.4 (0.5–21.2)	–	–	4.7 (0.8–22.8)	–	3.1 (0.6–14.5)
Clinical pharmacy	–	4.8 (0.4–41.5)	–	1.4 (0.3–5.8)	–	1.8 (0.5–6.2)
Pharmaceutical consultation	10.9 (1.6–48.4)	2.0 (0.1–21.6)	66.0 (19.1–94.1)	16.0 (6.2–35.4)	–	17.1 (6.8–36.9)
Pharmaceutical guidance	24.3 (10.4–47.2)	91.3 (53.0–99.0)	4.6 (0.4–34.9)	25.6 (9.6–52.6)	64.6 (12.9–95.7)	37.9 (17.2–64.2)
Pharmaceutical assistance	–	–	–	6.5 (1.7–21.6)	–	4.0 (1.1–13.9)
Importance (n = 79)						
Very important	95.9 (75.4–99.5)	98.0 (78.4–99.9)	90.8 (56.9–98.7)	83.2 (62.1–93.8)	35.4 (4.3–87.1)	85.5 (69.6–93.8)
Important	4.1 (0.5–24.6)		9.2 (1.3–43.1)	16.8 (6.2–37.9)	64.6 (12.9–95.7)	14.2 (6.0–30.0)
Not important	–	2.0 (0.1–21.6)	–	–	–	0.4 (0.1–2.8)

Source: PNAUM – Services, 2015.

The main reasons alleged by pharmacists for not performing such activities were: never having been requested, not disposing of the physical space, and not having enough time. Other mentioned reasons include the absence of specific training, lack of encouragement by the city, absence of physical structure, insufficient personnel on the pharmacy, among others.

The main names assigned to these activities were: “pharmaceutical guidance” (Northeast and South) and “pharmaceutical care” (North and Southeast). In the Midwest region, the most frequent name was “pharmaceutical consultation”; the name “pharmaceutical assistance” was observed only in the Southeast; and “clinical pharmacy,” only in the South and Southeast. It was not possible to investigate the explaining factors of these differences.

The most frequent functions performed at the same time with activities of clinical nature were: dispensing of medicines (93.6%), technical responsibility by the pharmacy (90.5%), supervision of other employees of the pharmacy (74.5%), and activities with the health team (61.2%).

In the Northeast, 100% of the pharmacists who claimed to perform activities of clinical nature reported they also perform dispensing of medicines, while in the Midwest, dispensing was declared only by 32.9% of the pharmacists who performed clinical activities. When questioned about the importance they assigned to such activities, most pharmacists who performed activities of clinical nature considered them very important (85.5%) or important (14.2%).

According to Table 3, the conditions and ways of conducting the pharmaceutical activities of clinical nature varied between regions. Most pharmacists said they perform such activities mainly along with physicians and nurses. Among all the pharmacists interviewed in the study, 1/4 of them stated they rarely participate in meetings with the health team and 1/5 said they have never participated (Table 5).

**Table 3:** Characterization of the activities of clinical nature performed by pharmacists of primary health care, according to the regions of Brazil. National Survey on Access, Use and Promotion of Rational Use of Medicines, 2015. (n = 79)

Dimension/Variable	North	Northeast	Midwest	Southeast	South	Brazil
					
	% (IC95%)	% (IC95%)	% (IC95%)	% (IC95%)	% (IC95%)	% (IC95%)
To whom they are offered						
All users	32.0 (14.5–56.6)	85.4 (39.6–98.1)	12.0 (1.7–51.3)	49.5 (26.4–72.9)	4.0 (0.4–32.5)	49.8 (27.9–71.8)
Users who require it	33.4 (13.3–62.2)	5.9 (0.6–38.9)	73.1 (27.5–95.1)	5.5 (1.6–17.6)	64.6 (12.9–95.7)	16.3 (6.4–35.5)
Users with difficulties in the use of medicines	30.5 (12.7–56.8)	6.7 (0.7–43.2)	12.6 (2.1–49.0)	28.8 (11.4–54.8)	27.3 (2.6–84.1)	23.0 (10.7–42.8)
Specific groups	4.1 (0.5–24.6)	2.0 (0.1–21.6)	2.3 (0.3–14.0)	16.2 (6.3–35.5)	4.0 (0.4–32.5)	10.9 (4.5–24.0)
Available resources						
Specific location	46.5 (23.6–70.9)	5.9 (0.6–38.9)	77.7 (33.5–96.0)	50.8 (27.6–73.7)	4.0 (0.4–32.5)	42.4 (22.4–65.3)
Registration system						
Computerized system	34.3 (14.5–61.6)	2.0 (0.1–21.6)	73.1 (27.5–95.1)	53.6 (29.5–76.1)	68.6 (14.6–96.5)	45.1 (24.7–67.2)
Specific registration filed in the pharmacy	50.5 (26.7–74.1)	8.7 (1.0–47.0)	83.7 (43.3–97.2)	38.5 (18.1–63.8)	96.0 (67.5–99.6)	40.3 (21.5–62.4)
User medical record	42.4 (20.6–67.7)	6.7 (0.7–43.2)	13.1 (2.0–52.4)	65.6 (37.9–85.7)	72.7 (15.9–97.4)	48.2 (26.9–70.1)
Other	3.4 (0.5–21.2)	–	–	6.2 (1.9–18.0)	68.6 (14.6–96.5)	4.0 (1.3–11.4)
Qualification and training	49.7 (26,1- 73,4)	98.0 (78,4 - 99,9)	89.5 (54.2–98.4)	56.5 (30.5–79.3)	8.0 (1.1–40.5)	65.2 (41.6–83.1)
Interaction with the health team						
Physicians	62.7 (35.9–83.5)	6.7 (0.7–43.2)	14.0 (2.4–51.7)	57.5 (31.9–79.6)	35.4 (4.3–87.1)	42.9 (23.7–64.4)
Nurses	54.6 (29.9–77.3)	8.7 (1.0–47.0)	14.0 (2.4–51.7)	57.9 (32.1–80.0)	35.4 (4.3–87.1)	43.0 (23.8–64.5)
Nutritionists	22.6 (7.1–52.6)	6.7 (0.7–43.2)	5.7 (0.7–34.3)	36.6 (17.4–61.3)	31.4 (3.5–85.4)	26.8 (13.2–47.0)
Dentists	50.5 (26.7–74.1)	4.8 (0.4–41.5)	5.7 (0.7–34.3)	29.6 (12.2–55.8)	31.4 (3.5–85.4)	23.8 (11.1–43.9)
Other	12.2 (3.7–33.1)	–	1.1 (0.1–11.1)	5.5 (2.4–12.2)	27.3 (2.6–84.1)	5.3 (2.5–11.2)

Source: PNAUM – Services, 2015.

**Table 5:** Participation of pharmacists in other activities in primary health care, according to the regions of Brazil. National Survey on Access, Use and Promotion of Rational Use of Medicines, 2015. (n = 285)

Dimension/Variable	North	Northeast	Midwest	Southeast	South	Brazil
					
	% (IC95%)	% (IC95%)	% (IC95%)	% (IC95%)	% (IC95%)	% (IC95%)
Participation in meetings of the health team						
Always	46.9 (32–62.3)	19.4 (4.3–56.1)	26.2 (12.5–46.9)	43.6 (28.5–59.9)	41.9 (23.6–62.6)	39.6 (29.2–51.0)
Repeatedly	4.6 (1.1–16.7)	–	–	5.4 (2.3–12.2)	2.1 (0.4–9.8)	3.8 (1.8–7.9)
Never	21.4 (10.8–38.0)	4.7 (1.3–15.8)	23.5 (9.2–48.2)	18.3 (9.8–31.4)	30.6 (12.8–57.0)	19.7 (12.7–29.2)
Sometimes	3.5 (0.8–13.9)	47.8 (11.3–86.9)	26 (9.2–54.9)	8.2 (3.4–18.5)	1.9 (0.3–12.9)	12.3 (6.1–23.0)
Rarely	23.6 (13.3–38.5)	28.1 (4.7–75.7)	24.3 (11.5–44.3)	24.6 (10.3–48.0)	23.5 (10.4–44.9)	24.7 (14.1–39.5)
Participation in other activities in the health units						
No	47.7 (32.9–93.0)	55.2 (15.8–89)	74.4 (54.1–87.8)	72.4 (59.0–82.7)	45.8 (25.8–67.1)	65.9 (55.5–75.0)
Yes	52.3 (37.0–67.1)	44.8 (11–84.2)	25.6 (12.2–45.9)	27.6 (17.3–41.0)	54.2 (32.9–74.2)	34.1 (25–44.5)
Activities						
With other sectors (education, social services, environment etc.)	63.4 (39.6–82.1)	36.4 (6.7–82.0)	71.3 (41.2–89.9)	61.9 (40.2–79.7)	67.7 (36.1–88.6)	61.2 (30.2–56.5)
Organizational activities of the community	44.1 (23.9–66.4)	12.0 (1.3–59.1)	77.6 (51.1–92)	37.6 (21.6–56.8)	40.4 (18.8–66.4)	38.8 (27–52.2)
Joint effort to solve problems in the community	67.8 (44.6–84.6)	41.4 (7.8–85.6)	89.1 (69.5–96.7)	32.1 (18.5–49.6)	44.5 (20.5–71.3)	42.9 (30.2–56.5)
Preservation of nature	21.4 (8.1–45.6)	2.1 (0.2–17.5)	36.6 (12.7–69.7)	24.0 (11.9–42.5)	38.0 (15.7–66.9)	25.8 (15.9–38.9)
Prevention and control of obesity	47.4 (26.4–69.4)	5.1 (0.5–35.1)	17.9 (3.5–56.9)	28.7 (15.0–47.9)	45.4 (21.2–72.0)	30.5 (19.8–43.9)
Physical activities	17.8 (5.6–44.2)	–	25.1 (7.1–59.8)	22.2 (11.6–38.5)	31.3 (13.1–57.9)	21.9 (13.8–33.0)
Prevention and control of hypertension and diabetes	80.2 (54.9–93.1)	39.4 (7.4–84.1)	90.9 (71–97.6)	75.0 (37.6–91.0)	71.2 (37.6–91.0)	71.5 (53.8–84.4)
Environmental control of diseases (e.g. combating dengue fever)	47.9 (27.0–69.7)	93.7 (73.6–98.8)	90.7 (68.7–97.8)	30.3 (16.9–48.2)	46.0 (21.6–72.4)	47.1 (33.5–61.2)
Prevention of cervical and uterine cancer	52.3 (30.4–73.3)	25.3 (4.0–73.1)	75.1 (45.6–91.6)	31.7 (18.0–49.5)	53.1 (26.8–77.8)	40.9 (28.6–54.4)
Prevention of prostate cancer	37.4 (18.7–60.7)	25.3 (4.0–73.1)	65.6 (35.1–87.1)	28.3 (15.4–46)	56.1 (28.9–80.1)	38.3 (26.3–51.8)
Prevention of sexually transmitted diseases	65.2 (41.4–83.2)	83.8 (40.5–97.5)	86.9 (55.7–97.2)	53.1 (32.9–72.3)	58.6 (30.6–81.9)	61.3 (46.2–74.5)
Family planning	69.8 (46.8–85.9)	9.2 (1.7–37.7)	42.8 (16.3–74.2)	38.4 (22.6–57.0)	33.8 (14.7–60.2)	36.3 (25.2–49.0)

Source: PNAUM – Services, 2015.

The study identified that, in Brazil, the registration of activities of clinical nature is mainly made in the user’s medical record, in a computerized system, and in a specific document filed at the pharmacy, in that order. The highest percentages of registration of these activities were found in the Southeast and Midwest. In the Northeast, the percentages of registration were negligible (2%; 8.7%; and 6.7%, respectively).

In Brazil, about half of the respondents reported that the pharmaceutical activities of clinical nature are offered to all users of the health unit. This was the most common answer in the Northeast region. In the Midwest, these activities are more often offered only to users who request them.

More than half of the interviewed pharmacists informed to have received some training.

According to Table 4, the provision of information by pharmacists about the use and storage of medicines at home, at the moment of delivery, varies between regions. The Midwest region stands out for providing these information, while the Northeast presents the lowest percentages. The significant majority of pharmacists states that they inform users on the use of medicines, and just over half informs on storage at home. Thus, the provision of information on use surpasses information on storage at home, which may indicate that pharmacists disregard the users’ lack of information about the care required to preserve the quality of the medicine.

**Table 4:** Information provided by pharmacists on the use and storage of medicines at the time of dispensing in primary health care, according to the regions of Brazil. National Survey on Access, Use and Promotion of Rational Use of Medicines, 2015. (n = 285)

Dimension/Variable	North	Northeast	Midwest	Southeast	South	Brazil
					
	% (IC95%)	% (IC95%)	% (IC95%)	% (IC95%)	% (IC95%)	% (IC95%)
Information on the use of medicines						
Always	93.1 (81–97.7)	71 (24.1–95.0)	97.6 (84.1–90.7)	76.1 (51.6–90.5)	82.3 (53.3–95)	79.5 (62.8–89.9)
Repeatedly	–	24.3 (3.2–75.6)	2.4 (0.3–15.9)	2.2 (0.9–5.3)	3.6 (0.8–13.9)	4.2 (1.5–11.4)
Sometimes	3.6 (1.1–11.0)	0.9 (0.1–7.8)	–	9.6 (3.9–22.1)	14.2 (3.0–47.0)	8.4 (3.0–17.1)
Rarely	3.2 (0.5–19.8)	0.9 (0.1–7.8)	–	12.1 (1.9–49.5)	–	7.6 (1.2–35.7)
Never	–	2.8 (0.5–14.5)	–	–	–	0.2 (0.1–1.1)
Information on the storage of medicines						
Always	59.2 (43.3–73.4)	23.5 (5.7–60.9)	67.7 (46.3–86.4)	58.6 (39.8–75.2)	53.8 (32.5–73.7)	55.8 (43–67.8)
Repeatedly	2.4 (0.3–15.5)		8.7 (2.0–31.1)	5.9 (3.0–11.3)	0.2 (0–1.8)	4.7 (2.6–8.2)
Sometimes	18.0 (9.0–32.6)	47.5 (11.1–86.8)	5.7 (2.0–14.9)	34.6 (18.1–56.0)	36.6 (18.8–59.1)	32.4 (20.3–47.5)
Rarely	5.7 (2.0–14.9)	24.3 (3.2–75.6)	8.5 (2.1–29.2)	0.6 (0.2–1.6)	1.6 (0.2–11.1)	3.8 (1.2–11.4)
Never	14.7 (6.1–31.3)	4.7 (1.1–17.4)	9.4 (3.5–23.1)		7.7 (1.1–38.7)	3.2 (1.4–7.2)

Source: PNAUM – Services, 2015.

Table 5 shows that, in Brazil, only about 1/3 of respondents reported participating in other activities carried out in the health unit. In general, the participation of pharmacists in these activities varies between regions. The Midwest presented the lowest percentage of participation of pharmacists in other activities in the health unit. Despite this, the percentages of participation in activities between them were more expressive when compared to other regions, except regarding the prevention and control of obesity. The Northeast is the region where pharmacists are less involved in family planning activities, and not at all in physical activity programs.

The fact that activities of prevention and control of obesity and those to encourage physical activities have less participation of pharmacists draws attention, as they are the ones that least require the use of medicines for being more related to lifestyle. However, we observed in Brazil a relevant participation of pharmacists in activities that involve other sectors and the community itself (Table 5), except in the Northeast. Such activities could be closer to health promotion.

## DISCUSSION

Although still incipient, activities of clinical nature occur in the Brazilian primary health care, but in unequal proportions in all regions.

In general, these activities require minimal resources for their accomplishment: conditions that ensure privacy in the pharmacist/patient therapeutic relationship, criteria for the provision of the service, documentation and training, among others^6^ ^,^ ^11^ ^,^ ^15^ ^,^ ^21^ ^,^ ^24^.

In this study, most pharmacists who claimed performing activities of clinical nature also stated not disposing of a specific place to perform them, which is an essential condition to the preservation of privacy and confidentiality in the activities with the user. Privacy can be defined as the personal desire to keep secret facts closely linked to the person; the secrecy of information ensures confidentiality. The assurance of privacy and confidentiality contributes to the therapeutic relationship to be more interactive and resolutive^6^ ^,^ ^17^.

We were able to identify a set of difficulties to the performance of these activities, according to the interviewed pharmacists. The reasons, in general, refer to structural problems or excess of activities under the responsibility of the pharmacist. These same problems were also reported by those who declared to perform activities of clinical nature concomitantly with other functions.

The incipient institutionalization of pharmaceutical activities of clinical nature in the primary healthcare of SUS, associated with the deficiencies regarding the implementation of PNAF ^4^, still limited in its guidelines, can explain the poor development of these activities. Although most pharmacists recognize them as important, only few are currently performing them.

In several countries, an important aspect of the activities of clinical nature concerns the organization of services with this purpose and the character of exclusive dedication by the person who performs these activities. Pharmacists who engage in these services are not authorized by the health systems or class authorities to carry out other activities, to prevent conflicts of interest, as well as to optimize the work they perform^11^ ^,^ ^21^ ^,^ ^24^.

Difficulties identified regarding the resources needed to carry out these activities, in the Brazilian primary health care, indicate some improvisation that demands efforts by professionals, besides not having criteria that allow rationalizing the provision of those activities on health services.

In several countries, qualification and training are requirements for performing any pharmaceutical activity of clinical nature. Both initial and periodical training are needed, considering the uniqueness of required skills and abilities^5^ ^,^ ^6^ ^,^ ^11^ ^,^ ^23^ ^,^ ^27^. Furthermore, the unmet need of specific training of the pharmacists hinders the understanding of the philosophical principles established for the activities of clinical nature, namely: the values, responsibilities, functions and activities, as well as the *modus operandi* of the process of care and management of these activities^6^ ^,^ ^13^ ^,^ ^19^ ^,^ ^27^.

Concerning the criteria for providing the service, according to the literature, the most used ones consider patients with higher risk of experiencing drug therapy problems and who present chronic medical conditions. This strategy directs resources and efforts to care for groups in which the impact of pharmaceutical interventions are larger^6^ ^,^ ^11^ ^,^ ^24^ ^,^ ^27^.

In this study, it was not possible to deepen the analysis of the various registration procedures of the activities of clinical nature. We observed that, in Brazil, the percentages of registration of the three modalities were very close, and it called our attention that, despite the Northeast being the region that most declared performing activities of clinical nature, the percentages of registration of these activities were insignificant there.

It should be stressed that the specific registration filed in the pharmacy may hinder the circulation of information among the professionals involved in the user’s care. The absence of registration indicates a low degree of institutionalization of these activities according to the philosophical premises required for a practice related to care, to meet the drug therapy needs of health services’ users^6^ ^,^ ^17^.

The registration of pharmaceutical interventions and the exchange of information among the professionals of the health team can contribute to the promotion of the safe and rational use of medicines. The registration of pharmaceutical activities of clinical nature systematizes the monitoring of the drug therapy employed by users and enables to evaluate the need, safety, and effectiveness of the use of medicines, which provides more favorable results during the drug therapy^6^ ^,^ ^18^.

The participation of different health professionals – among which is the pharmacist – in clinical meetings usually indicates a recognition, by the health team, of the place of each professional in the care provided to users. Interaction with other professionals in the health team occurs in several models of clinical pharmaceutical practice and it is recommended^6^ ^,^ ^12^ ^,^ ^19^.

The philosophy of pharmaceutical care ^2^ ^,^ ^6^ ^,^ ^17^ reinforces the need for this interaction, for understanding that it contributes to ensure the completeness of care. The interaction between professionals is essential for all the required resources and expertise to be available to solve the population’s health problems. Furthermore, care is also based in different interdisciplinary decision-making processes and on the complementarity of knowledge to promote the most appropriate care to people^6^.

In this study, the names assigned to the activities of clinical nature revealed that the expressions “pharmaceutical guidance” and “pharmaceutical care” are the most used between pharmacists who performed these activities. However, apart from the issue of naming, we identified in the respondents an understanding that the activities they carry out during dispensing would correspond to pharmaceutical activities of clinical nature. This may be related to the insufficient conceptual and philosophical discussion in Brazil on pharmaceutical care and to the disputes of the scientific field^2^ ^,^ ^19^ ^,^ ^16^ ^,^ ^22^ ^,^ ^26^.

In the Northeast, the fact that all pharmacists who claimed to perform activities of clinical nature also declared performing the dispensation of medicines stands out. Given that dispensing includes guidance to users, it is possible that these pharmacists understand that the activities of clinical nature they claim to perform are mainly dispensing, because the name most often attributed to those activities was “pharmaceutical guidance.”

Unlike the other regions, in the Midwest, dispensing was an activity reported by 32.9% of the pharmacists who claimed to develop activities of clinical nature. Since, in this region, the name most commonly attributed to the activities of clinical nature was “pharmaceutical consultation,” there may be, between these pharmacists, a clearer understanding of the nature of these activities and of dispensing.

Dispensing requires pharmacists to report, guide, and educate on the use of medicines. Such activity, particular to this professional – essential to the proper use of this technology – does not necessarily imply that the pharmacist who practices dispensing should take responsibility for the results of the patient’s drug therapy, which is required from those who perform the pharmaceutical care^2^ ^,^ ^6^ ^,^ ^12^ ^,^ ^15^ ^,^ ^17^ ^,^ ^21^.

In the Brazilian case, the choice for a definition of pharmaceutical care that includes the activities of dispensing, guidance, and education in health can hinder the understanding of what characterizes what is proper or innovative regarding activities of clinical nature^12^ ^,^ ^16^ ^,^ ^17^ ^,^ ^19^.

In this study, we observed little participation of pharmacists in educational and health-related activities in primary health care services, which indicates that this professional is not yet well integrated into the health team and that pharmaceutical services are still isolated from other health actions. The involvement of pharmacists in less medicalized activities^8^ can be related to the maintenance of the centrality of medicines in pharmaceutical practices.

With a view to promote the rational use of medicines, strategies that reduce the morbidity and mortality related to these technologies may be clinical in nature and may also include collective educational activities^20^. However, these actions cannot be confused with the pharmaceutical activities of clinical nature observed in other countries^2^ ^,^ ^17^ ^,^ ^19^.

This study raises some questions not yet investigated and others that require further development. It is important to investigate how the implementation of these activities occurred in pharmaceutical policies of other countries and in Brazil, which were the structuring elements of these clinical activities, and how the working process of pharmacists performing such activities in SUS takes place. We sought to identify the presence of philosophical elements, a process of care, and management aspects that could characterize what we decided to call, for the purposes of this study, pharmaceutical activities of clinical nature. However, it is necessary to signal the limitations of the results, which did not allow a better understanding of the regions and their pharmaceutical services, neither of the processes regarding the practices of clinical nature developed by the professional pharmacists.

The confrontation of drug-related morbidity and mortality^14^ has contributed to the reorganization of pharmaceutical practices. Although important pharmaceutical policies in Brazil have established pharmaceutical care as a guideline, it is necessary to undertake efforts to institutionalize these clinical activities, ensuring proper infrastructure, qualification of professionals, funding, and assessment of the results so that pharmacists and public administrators are encouraged to offer such activities in SUS, promoting the implementation of PNAF regarding all its guidelines.
